# On-treatment Comparative Effectiveness of Vitamin K Antagonists and Direct Oral Anticoagulants in GARFIELD-VTE, and Focus on Cancer and Renal Disease

**DOI:** 10.1055/s-0042-1757744

**Published:** 2022-11-03

**Authors:** Sylvia Haas, Alfredo E. Farjat, Karen Pieper, Walter Ageno, Pantep Angchaisuksiri, Henri Bounameaux, Samuel Z. Goldhaber, Shinya Goto, Lorenzo Mantovani, Paolo Prandoni, Sebastian Schellong, Alexander G.G. Turpie, Jeffrey I. Weitz, Peter MacCallum, Hugo ten Cate, Elizaveta Panchenko, Marc Carrier, Carlos Jerjes-Sanchez, Harry Gibbs, Petr Jansky, Gloria Kayani, Ajay K Kakkar, on behalf of the GARFIELD-VTE investigators

**Affiliations:** 1 Formerly Technical University of Munich, Munich, Germany; 2 Formerly Thrombosis Research Institute, London, United Kingdom; 3 Thrombosis Research Institute, London, United Kingdom; 4 Department of Medicine and Surgery, University of Insubria, Varese, Italy; 5 Department of Medicine, Ramathibodi Hospital, Mahidol University, Thailand; 6 Department of Medicine, University of Geneva, Switzerland; 7 Division of Cardiovascular Medicine, Brigham and Women's Hospital and Harvard Medical School, Boston, United States; 8 Department of Medicine (Cardiology), Tokai University School of Medicine, Japan; 9 Center for Public Health Research, University of Milan-Bicocca, Monza, Italy; 10 Arianna Foundation on Anticoagulation, Bologna, Italy; 11 Department of Health Sciences, Medical Department 2, Municipal Hospital Dresden, Germany; 12 McMaster University, Hamilton, Canada; 13 Department of Haematology, McMaster University and the Thrombosis and Atherosclerosis Research Institute, Hamilton, Ontario, Canada; 14 Queen Mary University of London, London, United Kingdom; 15 Department of Vascular Medicine and Internal Medicine, Maastricht University Medical Center and Cardiovascular Research Institute Maastricht; Maastricht, The Netherlands; 16 National Medical Research Center of Cardiology of Ministry of Health of the Russian Federation, Moscow, Russian Federation; 17 Department of Medicine, The Ottawa Hospital, Ottawa, Canada; 18 Tecnológico de Monterrey. Escuela de Medicina y Ciencias de la Salud., Monterrey, Mexico; 19 Instituto de Cardiología y Medicina Vascular, TecSalud, Sa Pedro Garza Garcia, Mexico; 20 Vascular Laboratory, The Alfred Hospital, Melbourne, Australia; 21 Motol University Hospital, Department of Cardiovascular Surgery, Prague, Czech Republic

**Keywords:** venous thromboembolism, vitamin K antagonists, direct oral anticoagulants, on-treatment comparative effectiveness, anticoagulation

## Abstract

**Background** Direct oral anticoagulants (DOACs) provide a safe, effective alternative to vitamin K antagonists (VKAs) for venous thromboembolism (VTE) treatment, as shown via intention-to-treat comparative effectiveness analysis. However, on-treatment analysis is imperative in observational studies because anticoagulation choice and duration are at investigators' discretion.

**Objectives** The aim of the study is to compare the effectiveness of DOACs and VKAs on 12-month outcomes in VTE patients using on-treatment analysis.

**Methods** The Global Anticoagulant Registry in the FIELD - VTE (GARFIELD-VTE) is a world-wide, prospective, non-interventional study observing treatment of VTE in routine clinical practice.

**Results** In total, 8,034 patients received VKAs ( *n* = 3,043, 37.9%) or DOACs ( *n* = 4,991, 62.1%). After adjustment for baseline characteristics and follow-up bleeding events, and accounting for possible time-varying confounding, all-cause mortality was significantly lower with DOACs than VKAs (hazard ratio: 0.58 [95% confidence interval 0.42–0.79]). Furthermore, patients receiving VKAs were more likely to die of VTE complications (4.9 vs. 2.2%) or bleeding (4.9 vs. 0.0%). There was no significant difference in rates of recurrent VTE (hazard ratio: 0.74 [0.55–1.01]), major bleeding (hazard ratio: 0.76 [0.47–1.24]), or overall bleeding (hazard ratio: 0.87 [0.72–1.05]) with DOACs or VKAs. Unadjusted analyses suggested that VKA patients with active cancer or renal insufficiency were more likely to die than patients treated with DOAC (52.51 [37.33–73.86] vs. 26.52 [19.37–36.29] and 9.97 [7.51–13.23] vs. 4.70 [3.25–6.81] per 100 person-years, respectively).

**Conclusion** DOACs and VKAs had similar rates of recurrent VTE and major bleeding. However, DOACs were associated with reduced all-cause mortality and a lower likelihood of death from VTE or bleeding compared with VKAs.

## Graphical abstract

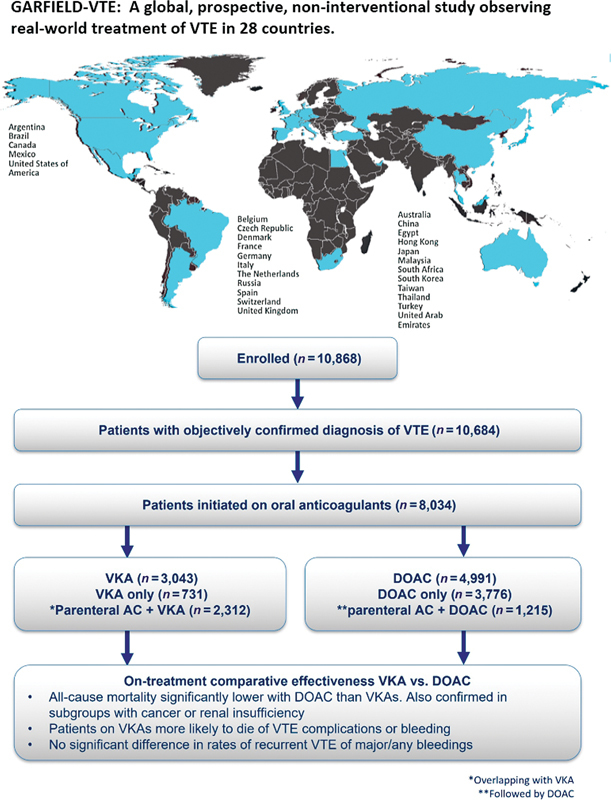

## Introduction

Anticoagulation (AC) is the mainstay of venous thromboembolism (VTE) treatment, with only select cases receiving thrombolytic or other reperfusion therapies. For many years, AC treatment consisted of a parenteral anticoagulant (such as heparin), overlapped with and followed by a vitamin K antagonist (VKA), such as warfarin. The introduction of direct oral anticoagulants (DOACs) provided a safe and effective alternative to this conventional treatment. 1 2 3 4 5 The changing landscape of VTE treatment requires observational studies to assess the comparative effectiveness of DOACs and VKAs in the global community setting. The Global Anticoagulant Registry in the FIELD – Venous Thromboembolism (GARFIELD-VTE), a prospective, multicenter, non-interventional, observational study of patients treated for acute VTE, 6 provides a framework for such a comparative effectiveness study.

Previous analyses of data from GARFIELD-VTE highlighted the high uptake of DOACs as an alternative AC treatment. 7 Patients were separated into five AC groups; those receiving parenteral AC alone, parenteral AC with a transition to VKAs, VKAs only, parenteral AC with a transition to DOACs, and DOACs only. We previously compared the effectiveness of VKAs and DOACs in an intention-to-treat (ITT) analysis, comparing the clinical outcomes of patients receiving these anticoagulants, with or without parenteral AC bridging. 8

ITT analysis avoids the bias associated with the non-random loss of participants. However, patients remain in the treatment group they received at baseline, regardless of whether they discontinued, crossed over to the other treatment(s) being studied, or adhered to the treatment over the course of follow-up. In contrast, on-treatment analysis is restricted to the period of follow-up during which a patient is on their assigned treatment.

On-treatment analysis is imperative in observational studies, because the duration and choice of AC are at the investigators' discretion and may change over time. We have seen that the length of time on treatment does vary compared to other indications, such as atrial fibrillation, where patients are on chronic use of drug indefinitely. CONSORT guidelines suggest that investigators should report both ITT and on-treatment analyses because “when both analyses provide identical conclusions, the confidence level of the investigator for the study results is augmented.” 9 This on-treatment analysis leads to essentially the same conclusions as our ITT analysis, thereby increasing the robustness of our previous results with the advantage that the same data base was used. Following the rules of good statistical practice for clinical research, refined methods were used for these analyses as described in the part of statistics. Available factors that are associated with the initial treatment decision, duration of treatment, and early discontinuation of treatment are considered in the modeling process.

The aim of this study was to compare the effectiveness of DOACs and VKAs (with or without parenteral AC bridging) on 12-month outcomes in VTE patients taking into account changes in treatment over time. Additional analyses focused on special patient populations with active cancer or renal insufficiency.

## Methods

### Study Design and Participants

A detailed description of the rationale and design of GARFIELD-VTE has been published previously. 6 The registry enrolled patients (≥18 years) between May 2014 and January 2017, diagnosed and treated across a range of care settings from 418 sites in 28 countries worldwide. The aim of the registry was to record local treatment practices; therefore, no specific treatments or procedures were mandated by the study protocol. Eligible patients were required to have an objective diagnosis of VTE (excluding superficial vein thrombosis) within 30 days of entry into the registry. Patients with recurrent VTE must have completed treatment for the previous event. Patients were excluded if long-term follow-up was not planned, or if they were participating in other studies that dictated visits, diagnostic procedures, or treatments.

### Selection of Study Sites

The national coordinating investigator identified the care settings they believed most accurately represented the management of VTE patients in their country. The contract research organization provided a list of sites that reflected these care settings, before contacting a random sample of sites for each care setting from the list. Sites that agreed to participate were recruited after a qualification telephone call. The investigator was required to complete a program providing guidance on patient screening, enrollment, and follow-up in the registry.

### Ethics Statement

The registry is conducted in accordance with the Declaration of Helsinki and guidelines from the International Conference on Harmonization on Good Clinical Practice and Good Pharmaco-epidemiological Practice, and adheres to all applicable national laws and regulations. Independent ethics committees for each participating country and the hospital-based institutional review boards approved the design of the registry. All patients provided written informed consent to participate and confidentiality and anonymity are maintained.

### Data Collection

Patient data relevant to VTE were collected through a review of clinical records and patient notes. Data were captured using an electronic case report form designed by eClinicalHealth Services, Stirling, United Kingdom, and submitted electronically via a secure website to the registry-coordinating center at the Thrombosis Research Institute, London, United Kingdom, which was responsible for checking the completeness and accuracy of data collected from medical records. The GARFIELD-VTE protocol requires that 10% of all electronic case report forms are monitored against source documentation, that there is an electronic audit trail for all data modifications, and that critical variables are subjected to additional audit. The data were extracted from the study database on October 14 ^th^ , 2020.

### Outcomes

The primary clinical outcomes were all-cause mortality, recurrent VTE, and major bleeding. Recurrent VTE was defined as a symptomatic event objectively confirmed by compression ultrasonography, contrast venography, computed tomography (CT) scan or magnetic resonance venography for deep vein thrombosis (DVT), and ventilation/perfusion scan, spiral CT scan, chest CT pulmonary angiography, or magnetic resonance angiography for PE. Major bleeding was defined according to the International Society on Thrombosis and Haemostasis criteria. 10 Non-major bleeding was defined as any overt bleeding not meeting the criteria for major bleeding. The rates of cancer, non-hemorrhagic stroke/transient ischemic attack, and myocardial infarction were also recorded. Additionally, information was collected regarding the cause of death and site of bleeding.

Patients were characterized as having active cancer if they were diagnosed and/or receiving treatment for their cancer during the window of ≤90 days before VTE diagnosis and up to 30 days after VTE diagnosis. Patients were defined as having a history of cancer if the cancer went into remission and the patient was not receiving any cancer treatment >90 days before the diagnosis of VTE. Cancer events that were diagnosed more than 30 days after the VTE diagnosis date were considered as cancer outcomes. Renal insufficiency was defined as stage III-V chronic kidney disease (moderate, severe, and kidney failure) in patients with a glomerular filtration rate of <60 mL/min/1.73 m ^2^ calculated with equation from Modification of Diet in Renal Disease (MDRD) study. 11

### Statistical Analysis

This study evaluates the comparative effectiveness of DOACs and VKAs with or without pretreatment with parenteral anticoagulants in VTE patients. Patients were excluded if they received parenteral AC alone, thrombolysis, or surgical or mechanical interventions. Patients are right censored when the treatment is completed or permanently discontinued. (OAC has been discontinued for more than 7 days. Discontinuations for less time are considered temporary discontinuations). This analysis used the “on treatment” or “per protocol” concept. 12 The effects of VKAs and DOACs were evaluated with marginal structural models using inverse probability weights (IPWs) adjusting for baseline characteristics, possible confounding by major bleeding events, and for informative censoring due to the effect of major bleeding on dropout. Baseline variables for the adjustment included: age, gender, ethnicity, body mass index (BMI), previous aspirin usage, VTE type (DVT alone, pulmonary embolism [PE] alone, DVT and PE), site of DVT (upper limb, lower limb, caval vein inferior or superior), care setting, physician specialty, treatment funding source, country, creatinine clearance, active cancer, recent bleeding or anemia, pregnancy, family history of VTE, history of cancer, known thrombophilia, prior VTE episodes, and renal insufficiency. Missing values for the adjustment were imputed using the Multivariable Imputation by Chained Equations (MICE) method. 13 Missing data were reported but not included in percentage calculations. Events were counted if they occurred within 365 days of the initial VTE diagnosis. Only the first occurrence of each event was considered.

Poisson regression was used to estimate unadjusted incidence rates (expressed per 100 person-years) and corresponding 95% confidence intervals (CI) by treatment for the clinical outcomes. Time-to-event analyses of outcomes were performed with IPWs time-varying Cox proportional hazards models. Variance of model coefficients was estimated using the Huber Sandwich Estimator. 14 The relationship between treatment groups was reported with hazard ratios (HRs) and their corresponding 95% CIs. All analyses are hypothesis generating and not conclusive in nature. Statistical analyses were conducted using R statistical software version 3.5.1. 15

## Results

### Patient Enrolment

**Fig. 1:**
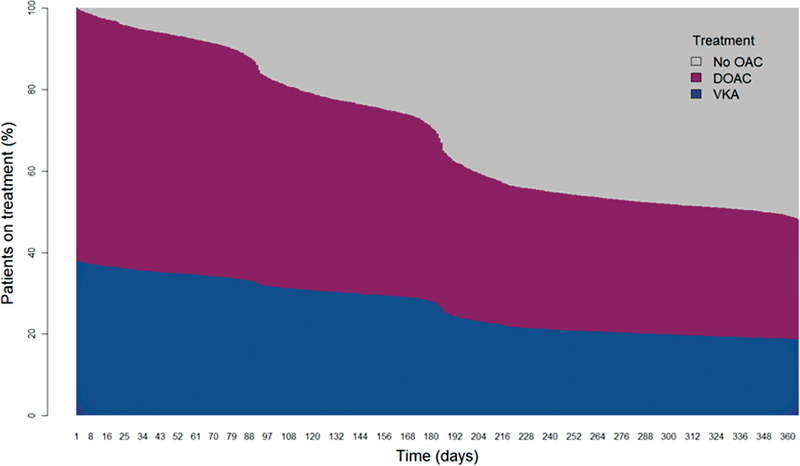
Treatment patterns over 12-months follow-up. No OAC includes end of study from death, termination of treatment or loss to follow-up. DOAC, direct oral anticoagulant; OAC, oral anticoagulant; VKA, vitamin-K antagonist.

Of the 11,840 VTE patients invited to enter the registry, 10,868 (91.8%) were enrolled. Of these, 184 patients were excluded because VTE was not objectively confirmed. Of the 10,684 patients eligible for this analysis, 8,034 were treated with oral anticoagulants with or without parenteral bridging; 4,991 (62.1%) received a DOAC and 3,043 (37.9%) received a VKA. Fig 1 illustrates the treatment pattern over 12-months follow-up. The median follow-up time was comparable for both treatment groups; VKA: 355 days (interquartile range [IQR]: 176–365) versus DOAC: 344 days (IQR: 141.5–365).

### Baseline Characteristics

Baseline characteristics are provided in Table 1 . The median age of patients receiving DOACs or VKAs was similar, 60 years (IQR:47–72) and 59 years (IQR:44–71), respectively, and a similar proportion were female (48.4 vs. 48.7%, respectively). DOACs were less frequently prescribed to Black patients than VKAs (1.7 vs. 10.7%), whereas Caucasian patients more frequently received DOACs (76.3 vs. 64.2%).

**Table 1:** Baseline characteristics

Variable	VKA ( *N* = 3,043)	DOAC ( *N* = 4,991)
Male, *n* (%)	1,560 (51.3)	2,576 (51.6)
Age, median (IQR)	59.0 (44.3, 70.7)	60.4 (46.7, 71.8)
Age groups, *n* (%)		
< 50	1,026 (33.7)	1,530 (30.7)
50–65	890 (29.2)	1,469 (29.4)
65–75	612 (20.1)	1,076 (21.6)
75–85	400 (13.1)	733 (14.7)
> 85	115 (3.8)	183 (3.7)
Ethnicity, *n* (%)		
Asian	438 (14.9)	793 (17.1)
Black	315 (10.7)	78 (1.7)
Caucasian	1,882 (64.2)	3,536 (76.3)
Other	297 (10.1)	229 (4.9)
Missing	111	355
BMI, median (IQR)	27.9 (24.3, 32.1)	27.5 (24.3, 31.6)
BMI categories		
Underweight (<18.5)	62 (2.3)	69 (1.5)
Normal (18.5–24.9)	756 (27.8)	1,296 (29.0)
Overweight (25–29.9)	911 (33.5)	1,639 (36.7)
Obese (≥30)	992 (36.5)	1,468 (32.8)
Missing	322	519
Creatinine clearance, mL/min, median (IQR)	93.7 (64.1, 127.3)	94.8 (68.1, 123.8)
Creatinine clearance, mL/min, *n* (%)		
I – Normal (≥90)	840 (32.7)	1,288 (30.6)
II – Mild (60–89)	1,053 (41.0)	2,072 (49.3)
III – Moderate (30–59)	499 (19.4)	746 (17.7)
IV – Severe (15–29)	79 (3.1)	48 (1.1)
V – Failure (<15)	97 (3.8)	49 (1.2)
Missing	475	788
Smoking status, *n* (%)		
Never	1,775 (59.9)	2,930 (61.5)
Ex-smoker	687 (23.2)	1,039 (21.8)
Current smoker	499 (16.9)	798 (16.7)
Missing	82	224
Care setting, *n* (%)		
Hospital	2,230 (73.3)	3,474 (69.6)
Outpatient setting	813 (26.7)	1,517 (30.4)

Abbreviations: BMI, body mass index; DOAC, direct oral anticoagulants; IQR, interquartile range; VKA, vitamin K antagonists.

Table 2 summarizes all risk factors present in both patient groups. Patients receiving VKAs were more likely to have had acute medical illness (7.2 vs. 4.3%), or have been hospitalized within the 3 months preceding VTE diagnosis (12.1 vs. 9.7%) than those receiving DOACs. Chronic heart failure and a recent bleed or anemia were also more common in patients receiving VKAs (4.3 vs. 2.4% and 4.2 vs. 2.1%, respectively).

**Table 2:** VTE risk factors present within the 3 mo preceding VTE diagnosis

Risk factor, *n* (%)	VKA ( *N* = 3,043)	DOAC ( *N* = 4,991)
Acute medical illness a	218 (7.2)	214 (4.3)
Hospitalization a	367 (12.1)	483 (9.7)
Long-haul travelling a	168 (5.5)	286 (5.7)
Surgery a	345 (11.3)	617 (12.4)
Trauma of the lower limb a	212 (7.0)	443 (8.9)
Active cancer	144 (4.7)	264 (5.3)
Pregnancy	55 (1.8)	31 (0.6)
Recent bleed or anemia	129 (4.2)	106 (2.1)
Chronic heart failure	132 (4.3)	121 (2.4)
Chronic immobilization	183 (6.0)	206 (4.1)
Family history of VTE	192 (6.3)	354 (7.1)
History of cancer	285 (9.4)	493 (9.9)
Hormone replacement therapy (females)	46 (1.5)	82 (1.6)
Known thrombophilia	85 (2.8)	144 (2.9)
Oral contraception (females)	143 (4.7)	316 (6.3)
Prior episode of DVT and/or PE	514 (16.9)	795 (15.9)
Renal insufficiency	179 (5.9)	107 (2.1)

Abbreviations: DOAC, direct oral anticoagulant; DVT, deep vein thrombosis; VKA, vitamin K antagonist; VTE, venous thromboembolism.

a Provoking factors.

Patients with PE ± DVT were as likely to receive a DOAC or a VKA (40.3 vs. 36.9%), as those with DVT alone (59.7 vs. 63.1%). Of those with lower limb DVT, patients with isolated distal DVT were more likely to receive a DOAC than a VKA (38.8 vs. 29.5%), whereas patients with proximal (±distal) DVT were more likely to receive a VKA (70.5 vs. 61.2%) ( Table 3 ).

**Table 3:** VTE characteristics

Variable, *n* (%)	VKA ( *N* = 3,043)	DOAC ( *N* = 4,991)
VTE type		
DVT	1,920 (63.1)	2,978 (59.7)
PE	740 (24.3)	1,127 (22.6)
DVT and PE	383 (12.6)	886 (17.8)
Site of DVT		
Upper limb	86 (3.7)	180 (4.7)
Lower limb	2,184 (95.1)	3,630 (93.9)
Caval vein (inferior)	15 (0.7)	32 (0.8)
Caval vein (superior)	12 (0.5)	22 (0.6)
Type of lower limb DVT		
Distal	638 (29.5)	1,394 (38.8)
Proximal	956 (44.2)	1,143 (31.8)
Proximal & distal	570 (26.3)	1,057 (29.4)
Missing	879	1,397
Type of PE		
Main	308 (27.6)	560 (28.1)
Lobar	360 (32.3)	571 (28.6)
Segmental	338 (30.3)	677 (34.0)
Sub-segmental	109 (9.8)	186 (9.3)
Missing	1,928	2,997

Abbreviations: DOAC, direct oral anticoagulants; DVT, deep vein thrombosis; PE, pulmonary embolism; VKA, vitamin K antagonist; VTE, venous thromboembolism.

Patients enrolled from vascular medicine practices were more likely to receive a DOAC than a VKA (49.4 vs. 39.8%), whereas those enrolled from internal medicine practices were more likely to receive a VKA (51.1 vs. 37.3%). Patients enrolled in Europe were more likely to receive DOACs than a VKA (60.5 vs. 52.9%), whereas the opposite was true in patients enrolled in the Middle East and South Africa (6.6 vs. 19.5%). A breakdown of the number of patients from each country receiving DOACs or VKAs is provided in Supplementary Table S1 .

### Clinical Outcomes

**Fig. 2:**
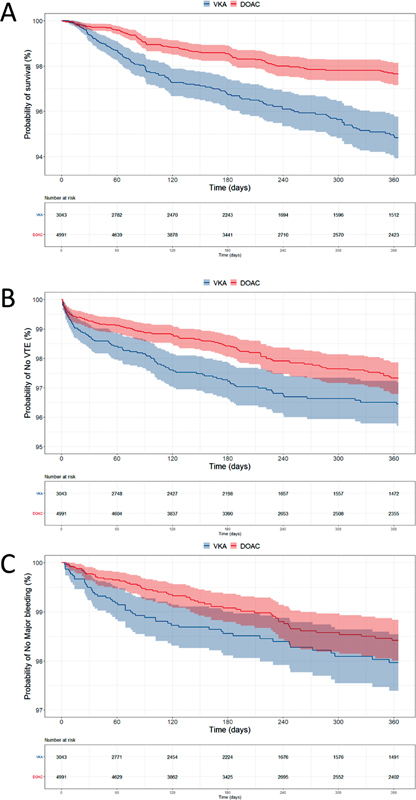
Kaplan-Meier curves for **(A)** all-cause mortality, **(B)** recurrent VTE, and **(C)** major bleeding. The number of patients at risk at each time point is shown below each curve.

After 12 months follow-up, the unadjusted rate of all-cause mortality was lower in patients receiving DOACs than in those receiving VKAs (2.61 [2.12–3.20] per 100 person-years vs. 5.69 [4.76–6.79] per 100 person-years). The rate of recurrent VTE was similar in patients receiving DOACs and VKAs (2.97 [2.44–3.61] per 100 person-years vs. 4.32 [3.52–5.30] per 100 person-years, respectively). The rate of major bleeding was also comparable (DOAC: 1.69 [1.30–2.18] per 100 person-years vs. VKA: 2.35 [1.78 − 3.10] per 100 person-years) ( Table 4 ). The unadjusted survival curves for all-cause mortality, recurrent VTE, and major bleeding are provided in Figs. 2A , B , and C , respectively.

**Table 4:** 12 Month unadjusted event rates. event rates are shown per 100 person-years

Outcome	VKA ( *N* = 3,043)		DOAC ( *N* = 4,991)	
	Number of events	Rate (95% CI)	Number of events	Rate (95% CI)
All-cause mortality	122	5.69 (4.76 − 6.79)	90	2.61 (2.12 − 3.20)
Recurrent VTE	91	4.32 (3.52 − 5.30)	101	2.97 (2.44 − 3.61)
Major bleeding	50	2.35 (1.78 − 3.10)	58	1.69 (1.30 − 2.18)
Any bleeding	258	12.65 (11.20 − 14.29)	395	12.02 (10.89 − 13.26)
Myocardial infarction	12	0.56 (0.32 − 0.99)	15	0.44 (0.26 − 0.72)
Stroke/TIA	8	0.37 (0.19–0.75)	21	0.61 (0.40 − 0.93)

Abbreviations: TIA, transient ischemic attack; VTE, venous thromboembolism.

**Fig. 3:**
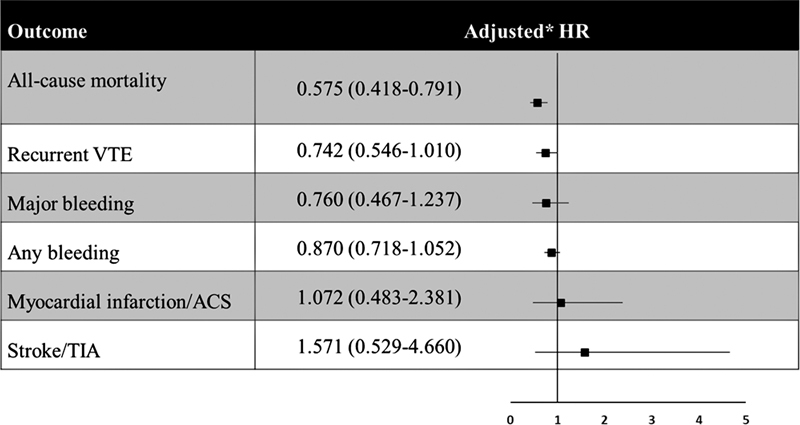
Adjusted hazard ratios between DOAC and VKA (reference) treatment groups. Values <1 favor DOAC treatment over VKA because they are indicative of a reduction in the hazard rate. * HRs were adjusted for major bleeding and dropout at follow-up in addition to the following baseline characteristics: age, gender, ethnicity, BMI, previous aspirin usage, VTE type (DVT alone, PE alone, DVT, and PE), site of DVT (upper limb, lower limb, caval vein inferior or superior), care setting, physician specialty, treatment funding source, country, creatinine clearance, active cancer, recent bleeding or anemia, pregnancy or postpartum, family history of VTE, history of cancer, known thrombophilia, prior VTE episodes, and renal insufficiency. DOAC, Direct oral anticoagulants; DVT, deep vein thrombosis; PE, pulmonary embolism.

After adjustment, the rate of all-cause mortality remained lower in patients receiving DOACs than in those receiving VKAs (HR: 0.58; 95% CI: 0.42–0.79, *p* = 0.001). The risk of recurrent VTE was comparable with DOACs and VKAs (HR: 0.74; 95% CI: 0.55–1.01), *p* = 0.06. The rates of major bleeding were similar in patients receiving DOACs and VKAs (HR: 0.76; 95% CI: 0.47–1.24, *p* = 0.270) as were the rates of myocardial infarction and stroke ( Fig 3 ). Patients receiving DOACs were less likely to die from VTE complications than those receiving VKAs (2.2 vs. 4.9%), but were more likely to have cancer-related deaths (45.6 vs. 34.4%). They were also less likely to have a fatal bleed than those receiving VKAs (0.0 vs. 4.9% of all deaths) ( Table 5 ). The site of recurrent DVT did not differ between treatment groups, however, the burden of PE seemed to be lower in the DOAC group. The main and lobar pulmonary branches were affected in 74.2% of the patients treated with VKAs versus 43.3% in the DOAC group ( Supplementary Table S2 ). The most common sites of major bleeding in patients receiving DOACs and VKAs were the upper gastrointestinal tract (15.5 and 10.0%), lower gastrointestinal tract (19.0 and 22.0%), and uterus (17.2 and 12.0%) ( Supplementary Table S3 ).

**Table 5:** Cause of death

Cause of death, *n* (%)	VKA ( *N* = 122)	DOAC ( *N* = 90)
VTE	6 (4.9)	2 (2.2)
Stroke	2 (1.6)	1 (1.1)
Cardiac	10 (8.2)	11 (12.2)
Cancer-related	42 (34.4)	41 (45.6)
Bleed	6 (4.9)	0 (0.0)
Other	31 (25.4)	14 (15.6)
Unknown	25 (20.5)	21 (23.3)

Abbreviation: VTE, venous thromboembolism.

In patients with renal insufficiency, the unadjusted rate of all-cause mortality was lower in patients receiving DOACs than in those receiving VKAs (4.70 [3.25–6.81] per 100 person-years vs. 9.97 [7.51–13.23] per 100 person-years). The rates of recurrent VTE and major bleeding were comparable between treatment groups ( Table 6 ). In patients with concomitant active cancer, the rate of all-cause mortality was lower in those treated with DOACs than in those treated with VKAs (26.52 [19.37–36.29] per 100 person-years vs. 52.51 [37.33–73.86] per 100 person-years). The rate of recurrent VTE was also lower in cancer patients receiving DOACs than in those receiving VKAs (3.40 [1.42–8.18] per 100 person-years vs. 17.93 [9.93–32.38] per 100 person-years). The rates of major bleeding were comparable between treatment groups ( Table 7 ).

**Table 6:** 12 Month unadjusted event rates in VTE patients with renal insufficiency

Outcome	VKA ( *N* = 675)		DOAC ( *N* = 843)	
	Number of events	Rate (95% CI)	Number of events	Rate (95% CI)
All-cause mortality	48	9.97 (7.51–13.23)	28	4.70 (3.25–6.81)
Recurrent VTE	28	5.96 (4.11–8.63)	16	2.73 (1.67–4.46)
Major bleeding	18	3.79 (2.39–6.01)	17	2.87 (1.79–4.62)
Any bleeding	65	14.18 (11.12–18.09)	89	15.92 (12.93–19.59)
Myocardial infarction	6	1.25 (0.56–2.79)	3	0.50 (0.16–1.57)
Stroke/TIA	5	1.04 (0.43–2.50)	2	0.34 (0.08–1.34)

Abbreviations: TIA, transient ischemic attack; VTE, venous thromboembolism.

**Table 7:** 12 month unadjusted event rates in VTE patients with active cancer

Outcome	VKA ( *N* = 144)		DOAC ( *N* = 264)	
	Number of events	Rate (95% CI)	Number of events	Rate (95% CI)
All-cause mortality	33	52.51 (37.33 − 73.86)	39	26.52 (19.37 − 26.29)
Recurrent VTE	11	17.93 (9.93 − 32.38)	5	3.40 (1.42 − 8.18)
Major bleeding	7	11.24 (5.36 − 23.58)	6	4.10 (1.84 − 9.12)
Any bleeding	17	28.05 (17.44 − 45.13)	26	18.59 (12.66 − 27.30)
Myocardial infarction	2	3.18 (0.80 − 12.73)	0	N/A
Stroke/TIA	0	N/A	2	1.36 (0.34 − 5.44)

Abbreviations: TIA, transient ischemic attack; VTE, venous thromboembolism.

### Time on Treatment

When comparing the median (Q1,Q3) time in days that patients were on treatment, patients that were treated with VKA were on treatment for longer than those treated with DOAC: 355.0 (176.0, 365.0) versus 344 (141.5, 365.0) ( Table 8 ). It should also be noted that patient follow-up was stopped at the time the treatment ended so the time of follow-up is identical to the time on treatment.

**Table 8:** Follow-up time by treatment

Follow-up (days)	VKA ( *N* = 3,043)	DOAC ( *N* = 4,991)	Total ( *N* = 8,034)
Mean (SD)	257.4 (122.7)	252.7 (123.9)	254.5 (123.4)
Median (Q1,Q3)	355.0 (176.0, 365.0)	344.0 (141.5, 365.0)	346.0 (157.0, 365.0)
Min–Max	1.0–365.0	1.0–365.0	1.0–365.0
Missing	0	0	0

Abbreviations: DOAC, direct oral anticoagulants; VKA, vitamin K antagonist.

## Discussion

Garfield-VTE was launched shortly after the clinical introduction of DOACs. Differences in ethnicity and geography were observed between the two oral AC groups. Geographical differences observed between treatment patterns with DOACs or VKAs may reflect the availability or approval status for DOACs in the respective countries worldwide. These differences may also represent inherent global health inequities and reimbursement differences for DOACs versus VKAs.

This on-treatment comparative effectiveness analysis of VKAs and DOACs demonstrates that the risk of all-cause mortality in VTE patients is more than one-third lower with DOACs than with VKAs. Both fatal bleeds and VTE-related deaths were reduced in patients receiving DOACs. Our findings of significant reduction in VTE-related deaths in the DOAC group are consistent with the findings of Mai et al, who performed a meta-analysis of randomized clinical trials (RCTs) evaluating the effect of extended AC as secondary prevention for VTE compared with placebo. The authors found that DOACs were associated with a reduction in overall (risk ratio [RR], 0.48; 95% CI, 0.27–0.86; *p* = 0.01) and VTE-related (RR, 0.36; 95% CI, 0.15–0.89; *p* = 0.03) mortality, whereas VKAs were not. 16 This meta-analysis also described that VKAs and DOACs similarly prevented recurrent VTE, 16 which we can confirm through our real-world observations, i.e. the risk of recurrent VTE, as well as arterial events such as myocardial infarction and stroke, was not significantly different between treatment groups in GARFIELD-VTE. Regarding arterial events, however, it must be noted that the event rates in both groups are very low and unlike venous events, these arterial events are based on clinical information provided by the investigator and rather than objectively proven diagnoses. In contrast to Mai et al 16 we could not confirm a general reduction of bleeding (both major and overall) in favor of DOACs. However, our findings of reduced fatal bleedings in the DOAC group should be reemphasized.

The RCTs of apixaban, dabigatran, edoxaban, and rivaroxaban showed comparable rates of all-cause mortality between DOACs and VKAs. 1 2 3 4 Our results of reduced mortality are in agreement with the non-interventional XALIA study programme that showed a significant reduction in mortality with rivaroxaban compared with conventional AC treatment. 17 Indeed, due to the early clinical availability of rivaroxaban worldwide, approximately 80% of patients receiving a DOAC were prescribed rivaroxaban in GARFIELD-VTE. 7 The mortality results of our comparative effectiveness analyses also concur with a recent meta-analysis of real-world studies comparing effectiveness and safety of the DOACs rivaroxaban and apixaban with standard of care in patients with VTE. The authors showed that in real-world practice, rivaroxaban and apixaban were associated with a lower risk of recurrent VTE and major bleeding events compared with standard of care and a survival benefit in patients treated with rivaroxaban was also observed. 18 Our findings are also in agreement with those of the START2-Register, which showed significantly reduced mortality in elderly VTE patients receiving DOACs compared with VKAs. However, the average age was significantly higher in that registry than in patients in GARFIELD-VTE. 19

The results of this on-treatment analysis are in agreement with our previous ITT analysis, which, after adjustment, estimated a 27% reduction in the risk of all-cause mortality with DOACs compared with VKAs. 8 We now report a 42% reduction in this study, using marginal structural models to control for time-varying confounding. This finding suggests that the benefits of DOACs over VKAs for VTE treatment may be greater than initially thought. Indeed, ITT analysis typically underestimates the superiority effect of a treatment. 20 On-treatment analyses are most informative in observational studies, because the choice and duration of AC are not dictated by a protocol, but are decided individually by the investigator and the patient. 21 This on-treatment analysis shows that the reduction in the risk of all-cause mortality remains after accounting for changes in the anticoagulant received or delivered, treatment non-adherence, or medically indicated discontinuation.

In contrast to RCTs, the GARFIELD-VTE registry includes patients with multiple comorbidities, including renal insufficiency and active cancer, who would have been excluded from the pivotal trials. We observed that the reduced rate of all-cause mortality with DOACs compared with VKAs was maintained in these vulnerable sub-groups. The analysis in VTE patients with active cancer is of particular interest because guidelines for the treatment of such patients changed during the course of patient follow-up in GARFIELD-VTE. Parenteral AC was the standard of care at the time of patient recruitment. Guidelines changed following the publication of the results of randomized trials comparing DOACs with dalteparin for VTE treatment in patients with active cancer. 22 23 24 DOACs are now often used instead of parenteral AC, but our subgroup comparison of DOACs and VKAs demonstrated that oral AC with VKAs would not be a reasonable alternative for patients with active cancer. When compared with VKAs the rates of recurrent VTE were lower with DOACs than with VKAs in this patient population.

Although the adjusted HRs for both major and overall bleeding favored DOAC treatment, differences were not statistically significant. In contrast, a meta-analysis of randomized controlled trials compared DOACs with VKAs for VTE treatment reported a 40% reduction in major bleeding with DOACs. 25 A potential explanation for this discrepancy is the fact that unlike the randomized trials, GARFIELD-VTE did not exclude patients at risk for bleeding, such as those with renal insufficiency or active cancer. A Japanese study that compared DOACs with VKA in the chronic phase of VTE treatment identified active cancer as an independent risk factor for major bleeding and recurrent VTE in the VKA group only but not the DOAC group. They concluded that DOACs appear to be an attractive therapeutic option for extended treatment of cancer-associated VTE. 26 In our analysis, there were no fatal bleeds in patients receiving DOACs, compared with six fatal bleeds in patients receiving a VKA (4.9% of all VKA-associated deaths).

Our study has limitations. As in any non-randomized study, there may be an imbalance in non-adjustable confounders which may have an impact on clinical outcome, including the cost and access to anticoagulants in each country. Furthermore, adjusted analyses were not carried out for subgroups due to an inadequate number of events. An additional limitation is the lack of central adjudication of outcome events and missing data, specifically on the causes of death. Finally, the majority of patients receiving DOACs within GARFIELD-VTE received rivaroxaban because this was the first DOAC in the market and the only one available when GARFIELD-VTE was launched. Therefore our results may not be generalizable to all DOACs.

## Conclusion

Our findings add to the growing body of evidence that supports DOACs over VKAs for VTE treatment because they are associated with reduced all-cause mortality, even in patients with active cancer or renal impairment. This is in addition to the convenience of fixed dosing without the need for coagulation monitoring.

## Notes

**What Is Known on This Topic?**

- Intention-to-treat comparative effectiveness analysis within the GARFIELD-VTE registry of real-world patients showed that DOACs provide a safe and effective alternative to VKAs for the treatment of VTE.
- Intention-to-treat analysis assesses all enrolled participants according to the treatment group assigned at baseline. It does not, however, account for patient treatment status over time (e.g., complete, incomplete, and altered treatment plan).
- CONSORT guidelines recommend both intention-to-treat and on-treatment evaluation.
- The on-treatment analysis accounts for alterations in treatment choice and plan over time.

**What Does This Paper Add?**

- This study provides an on-treatment comparative effectiveness analysis of DOACs and VKAs in VTE patients.
- At 12 months, rates of recurrent VTE, major bleeding, and overall bleeding with DOACs and VKAs are comparable.
- All-cause mortality was significantly lower with DOACs than with VKAs. Mortality related to VTE or bleeding was more likely with VKAs than DOACs.
- Unadjusted analyses suggested that VKA patients with active cancer or renal insufficiency were more likely to die than patients treated with DOAC.

## Footnotes

* *A full list of GARFIELD-VTE contributors is provided in the appendix* .

## References

[1] Hokusai-VTE Investigators Büller H R Décousus H Grosso M A Edoxaban versus warfarin for the treatment of symptomatic venous thromboembolism N Engl J Med 2013 369 15 1406 1415 23991658 10.1056/NEJMoa1306638

[2] RE-COVER Study Group Schulman S Kearon C Kakkar A K Dabigatran versus warfarin in the treatment of acute venous thromboembolism N Engl J Med 2009 361 24 2342 2352 19966341 10.1056/NEJMoa0906598

[3] EINSTEIN Investigators Bauersachs R Berkowitz S D Brenner B Oral rivaroxaban for symptomatic venous thromboembolism N Engl J Med 2010 363 26 2499 2510 21128814 10.1056/NEJMoa1007903

[4] AMPLIFY Investigators Agnelli G Buller H R Cohen A Oral apixaban for the treatment of acute venous thromboembolism N Engl J Med 2013 369 09 799 808 23808982 10.1056/NEJMoa1302507

[5] SOX trial investigators Kahn S R Shapiro S Wells P S Compression stockings to prevent post-thrombotic syndrome: a randomised placebo-controlled trial Lancet 2014 383(9920):880 888 24315521 10.1016/S0140-6736(13)61902-9

[6] Weitz J I Haas S Ageno W Global anticoagulant registry in the field - venous thromboembolism (GARFIELD-VTE). Rationale and design Thromb Haemost 2016 116 06 1172 1179 27656711 10.1160/TH16-04-0335

[7] Haas S Ageno W Weitz J I Anticoagulation therapy patterns for acute treatment of venous thromboembolism in GARFIELD-VTE patients J Thromb Haemost 2019 17 10 1694 1706 31220403 10.1111/jth.14548

[8] Bounameaux H Haas S Farjat A E Comparative effectiveness of oral anticoagulants in venous thromboembolism: GARFIELD-VTE Thromb Res 2020 191 103 112 32422442 10.1016/j.thromres.2020.04.036

[9] CONSORT Group Schulz K F Altman D G Moher D CONSORT 2010 statement: updated guidelines for reporting parallel group randomised trials PLoS Med 2010 7 03 e1000251 20352064 10.1371/journal.pmed.1000251 PMC2844794

[10] Subcommittee on Control of Anticoagulation of the Scientific and Standardization Committee of the International Society on Thrombosis and Haemostasis Schulman S Kearon C Definition of major bleeding in clinical investigations of antihemostatic medicinal products in non-surgical patients J Thromb Haemost 2005 3 04 692 694 15842354 10.1111/j.1538-7836.2005.01204.x

[11] National Kidney Foundation Foundation N K K/DOQI clinical practice guidelines for chronic kidney disease: evaluation, classification, and stratification Am J Kidney Dis 2002 39(2, suppl 1):S1 S266 11904577

[12] Tripepi G Chesnaye N C Dekker F W Zoccali C Jager K J Intention to treat and per protocol analysis in clinical trials Nephrology (Carlton) 2020 25 07 513 517 32147926 10.1111/nep.13709

[13] van Buuren S Groothuis-Oudshoorn K Mice: multivariate imputation by chained equations in R J Stat Softw 2011 45 03 1 67

[14] Joffe M Ten Have T Feldman H Kimmel S Model selection, confounder control, and marginal structural models: review and new applications Am Stat 2004 58 272 279

[15] Olié V Fuhrman C Chin F Lamarche-Vadel A Scarabin P Y de Peretti C Time trends in pulmonary embolism mortality in France, 2000-2010 Thromb Res 2015 135 02 334 338 25511577 10.1016/j.thromres.2014.12.002

[16] Mai V Guay C A Perreault L Extended anticoagulation for VTE: a systematic review and meta-analysis Chest 2019 155 06 1199 1216 31174635 10.1016/j.chest.2019.02.402

[17] Haas S Mantovani L G Kreutz R Anticoagulant treatment for venous thromboembolism: a pooled analysis and additional results of the XALIA and XALIA-LEA noninterventional studies Res Pract Thromb Haemost 2021 5 03 426 438 33870028 10.1002/rth2.12489 PMC8035798

[18] Wu O Morris S Larsen T B Effectiveness and safety of nonvitamin K oral anticoagulants rivaroxaban and apixaban in patients with venous thromboembolism: a meta-analysis of real-world studies Cardiovasc Ther 2022 2022 2.756682E6 10.1155/2022/2756682 PMC9203223 35801133

[19] coordinator of START2 Register Poli D Antonucci E Bertù L Very elderly patients with venous thromboembolism on oral anticoagulation with VKAs or DOACs: results from the prospective multicenter START2-Register Study Thromb Res 2019 183 28 32 31536872 10.1016/j.thromres.2019.08.024

[20] Weitkunat R Baker G Lüdicke F Intention-to-treat analysis but for treatment intention: how should consumer product randomized controlled trials be analyzed? Int J Stat Med Res 2016 5 90 98

[21] Ellenberg J H Intent-to-treat analysis versus as-treated analysis Drug Inf J 1996 30 02 535 544

[22] Hokusai VTE Cancer Investigators Raskob G E van Es N Verhamme P Edoxaban for the treatment of cancer-associated venous thromboembolism N Engl J Med 2018 378 07 615 624 29231094 10.1056/NEJMoa1711948

[23] Young A M Marshall A Thirlwall J Comparison of an oral factor Xa inhibitor with low molecular weight heparin in patients with cancer with venous thromboembolism: results of a randomized trial (SELECT-D) J Clin Oncol 2018 36 20 2017 2023 29746227 10.1200/JCO.2018.78.8034

[24] Caravaggio Investigators Agnelli G Becattini C Meyer G Apixaban for the treatment of venous thromboembolism associated with cancer N Engl J Med 2020 382 17 1599 1607 32223112 10.1056/NEJMoa1915103

[25] van Es N Coppens M Schulman S Middeldorp S Büller H R Direct oral anticoagulants compared with vitamin K antagonists for acute venous thromboembolism: evidence from phase 3 trials Blood 2014 124 12 1968 1975 24963045 10.1182/blood-2014-04-571232

[26] Wakakura S Hara F Fujino T Comparison of direct oral anticoagulants and warfarin in the treatment of deep venous thrombosis in the chronic phase Int Heart J 2018 59 01 126 135 29279522 10.1536/ihj.16-482

