# Supplementary material for: On-treatment Comparative Effectiveness of Vitamin K Antagonists and Direct Oral Anticoagulants in GARFIELD-VTE, and Focus on Cancer and Renal Disease
Source: TH Open. 2022 Nov 3;6(4):e354–64. doi: 10.1055/s-0042-1757744 (PMC9633227; doi:10.1055/s-0042-1757744)
Supplement: Supplementary file 1 — Supplementary Material [file 10-1055-s-0042-1757744-s22080036.pdf]

Supplementary Table S1 Anticoagulant treatment by country

Country	Total	VKA ($N = 3,043$)		DOAC ($N = 4,991$)	
	N	*n*	%	*n*	%
Argentina	139	85	61.2%	54	38.8%
Australia	286	33	11.5%	253	88.5%
Belgium	259	33	12.7%	226	87.3%
Brazil	75	49	65.3%	26	34.7%
Canada	446	118	26.5%	328	73.5%
China	216	68	31.5%	148	68.5%
Czech Republic	600	208	34.7%	392	65.3%
Denmark	200	68	34.0%	132	66.0%
Egypt	546	272	49.8%	274	50.2%
France	466	111	23.8%	355	76.2%
Germany	441	22	5.0%	419	95.0%
Hong Kong	68	17	25.0%	51	75.0%
Italy	473	110	23.3%	363	76.7%
Japan	116	11	9.5%	105	90.5%
Malaysia	109	62	56.9%	47	43.1%
Mexico	26	14	53.8%	12	46.2%
Netherlands	186	123	66.1%	63	33.9%
Russia	558	144	25.8%	414	74.2%
South Africa	366	319	87.2%	47	12.8%
South Korea	255	19	7.5%	236	92.5%
Spain	170	152	89.4%	18	10.6%
Switzerland	149	17	11.4%	132	88.6%
Taiwan	198	42	21.2%	156	78.8%
Thailand	170	159	93.5%	11	6.5%
Turkey	369	254	68.8%	115	31.2%
United Arab Emirates	11	3	27.3%	8	72.7%
United Kingdom	757	367	48.5%	390	51.5%
United States	379	163	43.0%	216	57.0%

Abbreviations: DOAC, direct oral anticoagulants; VKA, vitamin K antagonists; VTE, venous thromboembolism.

Supplementary Table S2 Cause of death

Cause of death, *n* (%)	VKA ($N = 122$)	DOAC ($N = 90$)
VTE	6 (4.9)	2 (2.2)
Stroke	2 (1.6)	1 (1.1)
Cardiac	10 (8.2)	11 (12.2)
Cancer-related	42 (34.4)	41 (45.6)
Bleed	6 (4.9)	0 (0.0)
Other	31 (25.4)	14 (15.6)
Unknown	25 (20.5)	21 (23.3)

Abbreviations: DOAC, direct oral anticoagulants; VKA, vitamin K antagonistsous thromboembolism.

 © 2022. The Author(s).

Supplementary Table S3 Site of VTE recurrence

Site of VTE recurrence, *n* (%)	VKA (*N* = 91)	DOAC (*N* = 101)
Site of DVT		
Upper limb	6 (9.2%)	6 (8.3%)
Lower limb	56 (86.2%)	63 (87.5%)
Caval vein (inferior)	3 (4.6%)	1 (1.4%)
Caval vein (superior)	0 (0.0%)	1 (1.4%)
Unusual site DVT	0 (0.0%)	1 (1.4%)
Missing	26	29
Pulmonary branch involved		
Main	10 (32.3%)	4 (13.3%)
Lobar	13 (41.9%)	9 (30.0%)
Segmental	6 (19.4%)	12 (40.0%)
Sub-segmental	2 (6.5%)	5 (16.7%)
Missing	60	71

Abbreviations: DOAC, direct oral anticoagulants; VKA, vitamin K antagonists; VTE, venous thromboembolism.

Supplementary Table S4 Site of major* bleeding

Site of bleed, *n* (%)	VKA (*N* = 50)	DOAC (*N* = 58)
GI lower	11 (22.0)	11 (19.0)
Uterine	6 (12.0)	10 (17.2)
GI upper	5 (10.0)	9 (15.5)
Intramuscular (no compartment syndrome)	4 (8.0)	0 (0.0)
Gingival	3 (6.0)	0 (0.0)
Epistaxis	2 (4.0)	1 (1.7)
Hemoptysis	2 (4.0)	0 (0.0)
Intraocular/Retinal	1 (2.0)	3 (5.2)
Macroscopic hematuria	1 (2.0)	5 (8.6)
Intramuscular (with compartment syndrome)	1 (2.0)	1 (1.7)
Skin (ecchymosis other than instrument site)	1 (2.0)	0 (0.0)
Hemothorax	1 (2.0)	0 (0.0)
Hemorrhagic stroke	1 (2.0)	3 (5.2)
Intra-spinal	0 (0.0)	0 (0.0)
Subconjunctival	0 (0.0)	0 (0.0)
Intra-articular	0 (0.0)	3 (5.2)
Intra-peritoneal	0 (0.0)	1 (1.7)
Retro-peritoneal	0 (0.0)	0 (0.0)
Puncture site	0 (0.0)	0 (0.0)
Hemopericardium	0 (0.0)	2 (3.4)
Other	7 (14.0)	4 (6.9)
Unknown	4 (8.0)	5 (8.6)

Abbreviations: DOAC, direct oral anticoagulants; VKA, vitamin K antagonists; VTE, venous thromboembolism.
*Major bleeding is defined as clinically overt bleeding that is associated with a fall in haemoglobin of 2g/dl or more or a transfusion of 2 or more units of unpacked red blood ells or whole blood or a critical site (intracranial, intraspinal, intraocular, pericardial, intra-articular, intramuscular with compartment syndrome, retroperitoneal), or a fatal outcome.

 © 2022. The Author(s).

Supplementary Material

A full list of GARFIELD-VTE principal investigators

Ab Loualidi, Abbey Wilcox, Abdul Parwani, Abdurrahim Colak, Abraham Bezuidenhout, Abu, Abdool-Carrim, Addala Azeddine, Adriaan Beyers, Adriaan Dees, Ahmed Mohamed, Ahmet Aksoy, Akihiko, Abiko, Akinori Watanabe, Alan Krichell, Alberto Alfredo Fernandez, Alberto Tosetto, Alexander Tsaregorodtsev, Alexey Khotuntsov, Alisha Oropallo, Alison Slocombe, Allan Kelly, Amanda Clark, Amr Gad, Amy Arouni, Andor Schmidt, Andrea Berni, Andrei D. Vishniagov, Andres Javier Kleiban, Andrew Machowski, Andrew Rapule Ratsela, Andrey Kazakov, Andrey L. Komarov, Angel Galvez, Ann Lockman, Anna Falanga, Anoop Chauhan, Antoni Riera-Mestre, Antonino Mazzone, Armando D'Angelo, Artur Herdy, Atsushi Kato, Attilia Pizzini, Ayman Abd Elhamid, Ebrahim Mahmoud Salem, Azlan Husin, Barbara Erdelyi, Barry Jacobson, Beatrice Amann-Vesti, Bektas Battaloglu, Benedicte Wilson, Benilde Cosmi, Bergmann Jean Francois, Berremeli Toufek, Beverley Hunt, Bhavesh Natha, Binali Mavitaş, Bisher Mustafa, Bonnie Chi Shan Kho, Boulon Carine, Brian Zidel, Brisot Dominique, Brousse Christophe, Bruno Trimarco, Canhua Luo, Carlos Alberto Cuneo, Carlos Jerjes Sanchez Diaz, Carsten Schwencke, Cas Cader, Celal Yavuz, Cesar, Javier Zaidman, Charles Lunn, Charlotte Bomken, Chau-Chung Wu, Cheng Hock Toh, Chern-En Chiang, Chevrier Elisa, Chien-Hsun Hsia, Chien-Lung Huang, Chi-Hang Kevin Kwok, Chih-Cheng Wu, Chi-Hung Huang, Chooi Kheng Chiew, Chris Ward, Christian Opitz, Christina Jeanneret-Gris, Chung Yin Ha, Chun-Yao Huang, Claude Luyeye Bidi, Clifford Smith, Cornelia Brauer, Corrado Lodigiani, Couturaud Francis, Cynthia Wu, Daniel Theodoro, Daniel Staub, Daniela Poli, David Keeling, David - Riesco Acevedo, David Scott, David Adler, David Jimenez, Davide Imberti, Desmond Creagh, Desmurs-Clavel Helene, Dirk Hagemann, Dirk Le Roux, Dirk Skowasch, Dmitry Belenky, Dmitry Dorokhov, Dmitry Petrov, Dmitry Zateyshchikov, Domenico Prisco, Dorthe Møller, Dr. Bárbara Pagán, Dusan Kucera, Ehab M. Esheiba, Elena Kochmareva, Elena N. Dankovtseva, Elizaveta Panchenko, Elkouri Dominique, Emre Dogan, Emre Kubat, Enrique Diaz, Eric Wai Choi Tse, Erik Yeo, Erman Hashas, Ernst Grochenig, Eros Tiraferri, Erwin Blessing, Escande Orthlieb Michèle, Esther Usandizaga, Ettore Porreca, Eva Lichnerova, Fabian Ferroni, Falvo Nicolas, Félix Ayala-Paredes, Fitjerald Henry, Franco Cosmi, Frans Erdkamp, Frederic Baumann, Gadel Kamalov, Gaetano Paparella, Garcia-Bragado Dalmau, Garrigues Damien, Garry Klein, Gaurand Shah, Geert Hollanders, Geno Merli, Georg Plassmann, George Platt, Georgy V. Smirnov, Germain Poirier, German Sokurenko, Ghassan Haddad, Gholam Ali, Giancarlo Agnelli, Gin Gan, Grace Kaye-Eddie, Gregoire Le Gal, Gregory Allen, Guillermo Antonio Llamas Esperón, Guillot Jean-Paul, Hagen Gerofke, Hallah Elali, Hana Burianova, Hans-Juergen Ohler, Haofu Wang, Harald Darius, Harinder S. Gogia, Harry Striekwold, Harry Gibbs, Hatice Hasanoglu, Hatice Turker, Hendrik Franow, Henri Bounameaux, Herbert De Raedt, Herman Schroe, Herman Sung Yu Liu, Hesham Salah ElDin, Hesham Zidan, Hiroaki Nakamura, Ho Young Kim, Holger Lawall, Hong Zhu, Hongyan Tian, Ho-Young Yhim, Hugo ten Cate, Hun Gyu Hwang, Hyeok Shim, Hyeon-Gyu Yi, Igor Kim, Igor Libov, Igor Sonkin, Igor Suchkov, Ik-Chan Song, Ilker Kiris, Ilya Staroverov, Irene Looi, Isabel M De La Azuela Tenorio, Ismail Savas, Ivan Gordeev, Ivo Podpera, Jae Hoon Lee, Jameela Sathar, James Welker, Jan Beyer-Westendorf, Jan Kvasnicka, Jan Van Meerbeeck, Jan Vanwelden, JangYong Kim, Jaromira Svobodova, Jaspal Gujral, Javier Tristan Galvar, Javier Claudio Marino, Jeannine Kassis, Jen-Yuan Kuo, Jhih-Yuan Shih, JiHyun Kwon, Jin Hyun Joh, Jin Hyun Park, Jin Seok Kim, Jinghua Yang, Jiri Krupicka, Jiri Lastuvka, Jiri Pumprla, Jiri Vesely, Joan Carlos Souto, João Antônio Correa, Johan Duchateau, John Perry Fletcher, Jorge del Toro, Jorge Guillermo Chavez Paez, Jørn Nielsen, Jose Dalmo Araujo Filho, Jose Maria Surinach, Jose Saraiva, Jose Antonio Diaz Peromingo, Jose Gomez Lara, Jose Luis Fedele, Joseph Chacko, Juan Carlos Álvarez Benitez, Juan Antonio Muntaner, Juan Moreno Hoyos Abril, Julian Humphrey, Julio Alberto Perez Sanchez, Julio Bono, Junji Kanda, Juree Boondumrongsagoon, Kai Hang Yiu, Kanchana Chansung, Karin Boomars, Kate Burbury, Katsuhiro Kondo, Kemal Karaarslan, Kensuke Takeuchi, Knut Kroeger, Konstantin Andreichuk, Konstantin Zrazhevskiy, Koscál Svatopluk, Kou-Gi Shyu, Kristel Vandenbosch, Kuan-Cheng Chang, Kuan-Ming Chiu, Kubina Jean-Manuel, Kwan Jing Wern, Kwo-Chang Ueng, Lalita Norasetthada, Laure Binet, Lee Ping Chew, Lei Zhang, Lidwine Tick, Lilia Beatriz Schiavi, Lily Lee Wong, Lisbeth Andersen, Louis Botha, Luc Capiau, Luc Timmermans Luciano Eduardo López, Luigi Ria, Luis Manuel hernandez Blasco, Luis Alberto Guzman, Luis Flota Cervera, Magnus Thorsen Jensen, Mahe Isabelle, Manuel Monreal Bosch, Manuel de los Rios Ibarra, Manuel Núñez Fernandez, Marc Carrier, Marc Righini, Marcelo Raul Barrionuevo, Marco Antonio Alcocer Gamba, Marco Cattaneo, Marco Moia, Margaret Bowers, Mariam Chetanachan, Mario Alberto Berli, Mark Fixley, Markus Faghih, Markus Stuecker, Marlin Schul, Martin Banyai, Martin Koretzky, Martin Myriam, Mary Elizabeth Gaffney, Masao Hirano, Masashi Kanemoto, Mashio Nakamura, Meng Lee Chang, Mersel Tahar, Messas Emmanuel, Michael Kovacs, Michael Leahy, Michael Levy, Michael Munch, Michael Olsen, Michel De Pauw, Michel Gustin, Michiel Van Betsbrugge, Mikhail Boyarkin, Miroslav Homza, Modise Koto, Mohamed Abdool-Gaffar, Mohamed Ayman Fakhry Nagib, Mohamed El- Dessoki, Mohamed Khan, Monniaty Mohamed, Moo Hyun Kim, Moon-Hee Lee, Mosaad Soliman, Mostafa Shawky Ahmed, Mostafa Soliman Abd el Bary, Moustafa A. Moustafa, Muhammad Hameed, Muhip Kanko, Mujibur Majumder, Nadezhda Zubareva, Natasha Roseva-Nielsen, Ngyuen Dang, Nicola Mumoli, Nik Azim Nik Abdullah, Nisa Makruasi, Nishen Paruk, Nonglak Kanitsap, Norberto Duda, Nordiana Nordin, Oksana A. Zemlianskaia, Ole Nyvad, Olga Barbarash, Orcun Gurbuz, Oscar Gomez Vilamajo, Oscar Martin Lopez Ruiz, Oscar Nandayapa Flores, Oscar Sanz

 © 2022. The Author(s).

Peláez, Ove Østergaard, Ozcan Gur, Pablo Javier Marchena, Pantep Angchaisuksiri, Patrick Carroll, Paul Coughlin, Pavel Lang, Peter Verhamme, Peter Baron von Bilderling, Peter Blombery, Peter MacCallum, Petr Jansky, Peuch Bernadette, Philippe De Vleeschauwer, Philippe Hainaut, Piera Maria Ferrini, Piriyaporn Iamsai, Pol Ravez, Ponchaux Christian, Pongtep Viboonjuntra, Ponlapat Rojnuckarin, Prahlad Ho, Pramook Mutirangura, Rachel Wells, Rafael Martinez, Raimundo Tirado Miranda, Ralf Kroening, Raquel Lopez Reyes, Raul Franco Diaz de Leon, Raymond Siu Ming Wong, Raz Alikhan, Reinhold Jerwan-Keim, Remedios Otero, Renate Murena-Schmidt, Reto Canevascini, Richard White, Richard Ferkl, Rika Van Herreweghe, Rita Santoro, Robert Mendes, Robert Klamroth, Robert Prosecky, Roberto Cappelli, Rudolf Spacek, Rupesh Singh, Sam Griffin, Sang Hoon Na, Sanjeev Chunilal, Saskia Middeldorp, Satoshi Nakazawa, Sebastian Schellong, See Guan Toh, Seinturier Christophe, Selim Isbir, Selma Raymundo, Serge Motte, Sergey Gryaznov, Serhat Erol, Serir Ozkan Aktogu, Servaas Donders, Seung Ick Cha, Seung-Hyun Nam, Sevestre Pietri Marie-Antoinette (NCI), Shaun Maasdorp, Shenghua Sun, Shenming Wang, Sherif Mohamed Essameldin, Sherif Mohamed Sholkamy, Shintaro Kuki, Shinya Goto, Shuichi Yoshida, Shunzo Matsuoka, Simon McRae, Simon Watt, Siong Leng Hon, Siriwimon Patanasing, Siwe-Nana Jean-Léopold, Somchai Insiripong, Somchai Wongkhantee, Soo-Mee Bang, Sophie Testa, Stanislav Zemek, Steffen Behrens, Stephan Dominique, Stuart Mellor, Suaran Singh Gurcharan Singh, Sudip Datta, Sunee Chayangsu, Susan Solymoss, Tamara Everington, Tarek Ahmed Adel Abdel-Azim, Tawatchai Suwanban, Taylan Adademir, Terence Hart, Terriat Béatrice, Thifhelimbilu Luvhengo, Thomas Bieri, Thomas Horacek, Thomas Zeller, Tim Reynolds, Tim Boussy, Tina Biss, Ting-Hsing Chao, Tomas Smith Casabella, Tomoya Onodera, Tontanai Numbenjapon, Victor Gerdes, Vladimir Cech, Vladimir Krasavin, Vladimir Tolstikhin, W.A. Bax, Wagih Fawzy Abdel Malek, Wai Khoon Ho, Walter Ageno, Walter Pharr, Weihong Jiang, Wei-Hsiang Lin, Weihua Zhang, Wei-Kung Tseng, Weimar Kunz Sebba Barroso de Souza, Wen-Ter Lai, Wilfried De Backer, Wilhelm Haverkamp, Winston Yoshida, Wolfgang Korte, Won Il Choi, Yana Kazachek, Yang-Ki Kim, Yasuhiro Tanabe, Yasushi Ohnuma, Yeung-Chul Mun, Yohan Balthazar, Yong Park, Yoshisato Shibata, Yulia N. Moiseeva, Zdenek Coufal, Zhenwen Yang, Zhicheng Jing, Zhicheng Jing, Zhongqi Yang.

 © 2022. The Author(s).
